# Differential Selection Effects of Continuous AITC Fumigation on Soil Microbial Communities and Functions and Identification of Tolerant Strains

**DOI:** 10.3390/microorganisms14020345

**Published:** 2026-02-02

**Authors:** Mengyuan Wang, Wenfeng Tian, Zhoubin Liu, Dongdong Yan, Yuan Li, Aocheng Cao, Qiuxia Wang, Wensheng Fang

**Affiliations:** 1 Engineering Research Center for Horticultural Crop Germplasm Creation and New Variety Breeding, Ministry of Education, Key Laboratory of Vegetable Biology of Hunan Province, College of Horticulture, Hunan Agricultural University, Changsha 410128, China; 2 State Key Laboratory for Biology of Plant Diseases and Insect Pests, Institute of Plant Protection, Chinese Academy of Agricultural Sciences, Beijing 100193, China

**Keywords:** allyl isothiocyanate, soil fumigation, soil microorganisms, sensitivity, tolerance

## Abstract

Allyl isothiocyanate (AITC) is effective as a bio-based fumigant in controlling soil-borne diseases; however, the selective pressure it exerts on soil microecology and evolutionary dynamics remains inadequately characterized. This study systematically investigated the remodeling effects of continuous AITC fumigation on soil microbial communities, functional genes, and functional strains by integrating metagenomic analysis and pure culture techniques. Results demonstrate that AITC drives directional selection from “sensitive” to “tolerant” microorganisms. Fungal communities exhibit greater cumulative damage than bacterial communities, with the proportion of significantly suppressed fungi increasing linearly from 9.3% at baseline to 35.7%. At the genus level, sensitive groups were predominantly enriched in pathogen-associated genera, e.g., *Pseudomonas* and *Xanthomonas*, whereas tolerant groups, represented by *Bacillus* and *Streptomyces*, maintained ecological dominance under continuous stress. Functionally, AITC induced differential evolution of functional gene repertoires. Nitrogen cycle genes (e.g., amoC) exhibited high negative sensitivity, with significant downregulation by 20%, whereas the TCA core module in the carbon cycle exhibited strong robustness. Virulence assays confirmed EC_50_ values for tolerant beneficial bacteria (*Bacillus* spp.) (>40 mg·L^−1^) were significantly higher than those for pathogens (1.3–7.9 mg/L). This study established a microbial “sensitive-tolerant” response framework under AITC stress, revealing the core potential of endogenous tolerant strains for the precise ecological restoration of fumigated soils.

## 1. Introduction

Allyl isothiocyanate (AITC), a naturally occurring organic sulfur compound that is abundant in cruciferous plants, includes horseradish and mustard. As an effective bio-based fumigant, AITC offers notable advantages, including ecological compatibility and low soil persistence. It effectively controls root-knot nematodes, soil-borne pathogens, and weeds through volatile toxicity, providing a key solution for addressing continuous cropping obstacles in intensive farming and advancing sustainable farming practices [1,2,3,4,5]. Compared to synthetic fumigants, AITC demonstrates superior environmental safety while achieving the specific biological targeting, making it a research focus in the field of biofumigation.

However, the potent oxidizing properties and broad-spectrum antimicrobial activity of AITC also induce significant ecological disturbances to soil microbial communities, with its detrimental effects being well-documented in multiple studies. Studies demonstrate that AITC application significantly reduces soil microbial diversity, with particularly pronounced and cumulative declines observed in fungal communities [6,7]. This non-selective antimicrobial action not only disrupts the balance of soil microbial communities but also substantially impairs the fundamental processes of soil nutrient cycling. Specifically, AITC significantly inhibits nitrification, reduces the abundance of key functional genes nitrogen fixation, nitrification, and denitrification (e.g., amoA and nirK), and substantially impedes microbial metabolic pathways related to carbon decomposition and phosphorus/sulfur transformation [6,8]. If this functional disruption is not promptly reversed, it will directly critically compromise the stability and functional services of soil environments.

Current strategies for restoring soil ecosystems after fumigation predominantly involve applying microbial inoculants or bio-organic fertilizers. Although existing remediation models aim to rebuild microbial barriers by adding broad-spectrum probiotics (e.g., *Bacillus* spp. and *Trichoderma* spp.) or fertilizers [9,10,11,12,13], they often lack specificity and precision. However, these approaches frequently overlook the evolutionary dynamics of indigenous microbial communities post-fumigation. Due to the inability to accurately distinguish between damaged (sensitive) and surviving (tolerant) communities, exogenous agents often exhibit unstable efficacy, owing to insufficient ecological niche competitiveness or environmental incompatibility [14,15]. This non-targeted and imprecise regulatory model constitutes a bottleneck for the rapid recovery of soil microecology after fumigation.

Therefore, elucidating the selective pressure of AITC fumigation on microbial communities and identifying the genetic profiles of sensitive versus tolerant taxa are fundamental prerequisites for precise microbial ecological remediation. This study utilizes metagenomic sequencing to systematically examine the dynamic effects of continuous AITC fumigation on the diversity and structure of soil bacteria, fungi, and pathogenic/beneficial microorganisms. Concurrently, it analyzes differential responses of functional genes associated with carbon, nitrogen, phosphorus, and sulfur cycling. By constructing detailed taxonomic profiles of AITC-sensitive and tolerant microorganisms, this study aims to provide scientific evidence for assessing AITC’s ecological footprint in soil ecosystems. Furthermore, it provides a robust theoretical foundation for developing precision remediation strategies based on functional compensation and advantageous immobilization principles.

This study aimed to systematically characterize the “sensitive-tolerant” profiles of soil microorganisms and functional genes under continuous AITC fumigation. We hypothesized that (1) continuous AITC fumigation exerts differential selective pressure, driving directional succession of soil microbial communities from sensitive to tolerant taxa rather than causing non-specific suppression; and (2) this selective pressure reshapes both the taxonomic composition and functional potential of the soil microbiome, with key biogeochemical cycles (e.g., nitrogen and carbon transformations) exhibiting distinct sensitivity patterns. Validating these hypotheses will facilitate the identification of endogenous AITC-tolerant beneficial strains, providing a scientific basis for precise ecological remediation of fumigated soils.

## 2. Materials and Methods

### 2.1. Experimental Design and Sample Collection

Five representative soil types were selected to encompass diverse microbiome backgrounds: Beijing loam (Cambisols), Harbin black soil (Phaeozems), Yunnan red soil (Ferralsols), paddy soil (Anthrosols), and greenhouse pepper soil (Technosols). Soils were sieved through a 2 mm mesh after removing stones and plant debris, then thoroughly homogenized. Soils were pre-incubated at 25 °C for 7 days to stabilize microbial activity. Soil moisture content was adjusted to 21% (*w*/*w*) prior to fumigation treatments. A 150 g aliquot of each soil was transferred into 500 mL glass bottles. Based on field trial results, the treatment group received 60 mg·kg^−1^ of 94% allyl isothiocyanate (AITC), while controls received an equal volume of sterile water [7]. Control samples were mock-fumigated (sealed identically but without AITC) to distinguish the chemical effect of AITC from the physical effects of the fumigation process. Each treatment combination included three independent biological replicates to ensure statistical reliability. Following a 7-day fumigation at 25 °C, samples were aerated for 24 h, after which the moisture content was readjusted. This fumigation cycle was repeated three times consecutively. Three consecutive fumigation cycles were implemented to evaluate long-term effects on microbial communities and soil functions, based on previous experimental designs [16]. After each fumigation, 30 g of soil samples were collected and stored at −80 °C for subsequent metagenomic analysis.

### 2.2. Soil Metagenomic Sequencing

Total genomic DNA was extracted from soil samples using the E.Z.N.A.^®^ Soil DNA Kit (Omega Bio-tek, Norcross, GA, USA). Following quality assessment, a paired-end library with 350 bp inserts was constructed and sequenced on the Illumina NovaSeq™ X Plus platform (Illumina, San Diego, CA, USA). Raw sequencing data were processed using fastp (v0.23.4; OpenGene, Shenzhen, China)for quality control, assembled with MEGAHIT (v1.1.2; The University of Hong Kong, Hong Kong, China), and open reading frames were predicted using Prodigal (v2.6.3; Oak Ridge National Laboratory, Oak Ridge, TN, USA). A non-redundant gene catalog was constructed with CD-HIT (v4.7; University of California, San Diego, CA, USA) at 90% identity and coverage thresholds. Gene abundance was quantified by mapping reads to the catalog using SOAPaligner (v2.21; BGI, Shenzhen, China). Taxonomic and functional annotations were assigned based on alignments to the NR (Non-Redundant) and KEGG (Kyoto Encyclopedia of Genes and Genomes) databases (Kyoto University, Kyoto, Japan) using DIAMOND (v2.0.13; University of Tübingen, Tübingen, Germany). Taxonomic and functional group abundances were determined by summing the abundances of their respective annotated genes.

### 2.3. Microbial Isolation and Tolerance Assessment

Target bacterial strains were isolated from repeatedly fumigated soil samples using standard pure culture techniques. One gram of triply fumigated fresh soil was serially diluted. Appropriately diluted aliquots were spread-plated on Nutrient Agar (NA; Beijing Obo Star Biotechnology, Beijing, China) and incubated at 30 °C for 48 h, purified through three to four rounds of subculturing on fresh NA plates. Genomic DNA was extracted using the M5 Hiper Bacterial Genomic DNA Extraction Kit (Juhemei, Beijing, China). The 16S rRNA gene was amplified with universal bacterial primers 338F/806R and sequenced at Shanghai Sangon Biotech. The resulting sequences were compared against the NCBI database using BLAST (Basic Local Alignment Search Tool; National Center for Biotechnology Information, Bethesda, MD, USA) to determine taxonomic affiliation.

The inhibitory activity of AITC against typical pathogens (*Fusarium oxysporum*, *F. graminearum*, and *Ralstonia solanacearum*) and beneficial microorganisms (*Trichoderma polysporum*, *Bacillus amyloliquefaciens*, *B. subtilis*, and *Sinomonas atrocyanea*) was evaluated using vapor-phase fumigation. For bacterial assays, suspensions were inoculated into LB liquid medium (Beijing Obo Star Biotechnology, Beijing, China) AITC volatilization was controlled using a filter paper diffusion method to establish concentration gradients ranging from 1 to 32 m·L^−1^. After 24 h of shaking incubation at 28 °C in darkness, OD_600_ was measured to calculate inhibition rates and specific growth rates during the logarithmic phase. Growth kinetics were monitored through periodic sampling over 0–68 h, with absorbance measurements used to construct growth curves and evaluate strain-specific tolerance to AITC. For fungal assays, the mycelial growth rate method was used. A 5 mm mycelial disk was inoculated at the center of a PDA plate (Qingdao Rishui Biotechnology, Qingdao, China). AITC was applied to a filter paper disk attached to the plate lid, and the plate was immediately sealed. Plates were incubated at 25 °C in the darkness for 2–6 days. When control colonies covered approximately two-thirds of the plate surface, diameters were measured using the cross-diameter method to calculate mycelial growth inhibition rates.

### 2.4. Statistical Analysis

Statistical analyses were performed using SPSS 26.0. Significant differences between groups were assessed with independent samples *t*-tests or one-way ANOVA, with Tukey’s HSD test applied for post hoc comparisons following ANOVA. Differences in alpha diversity indices were evaluated using the Wilcoxon signed-rank test. Beta diversity was visualized through principal coordinate analysis (PCoA) based on Bray–Curtis distances. Differences in overall community structure between groups were further assessed using analysis of similarities (ANOSIM). In line with previous methodologies [17], microbial taxa showing no significant change in abundance (*p* > 0.05) throughout the three consecutive fumigation cycles were classified as tolerant. Taxa demonstrating a significant response (*p* < 0.05), specifically to the first fumigation, were defined as sensitive. These were subsequently categorized into positive- and negative-response sensitive strains based on the direction of their abundance change.

## 3. Results

### 3.1. AITC Fumigation Reshapes Soil Microbial Community Diversity and Structure

**Figure 1:**
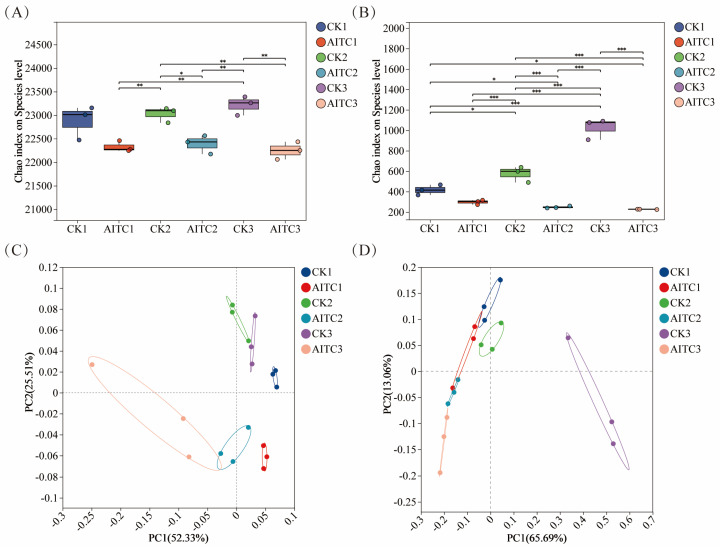
Differences in bacterial and fungal α-diversity indices and principal coordinate analysis (PCoA) based on the Bray–Curtis matrix. (**A**,**B**) Chao1 index of bacterial (**A**) and fungal (**B**) communities under different treatments. CKi: Control groups with i-times of mock fumigation (i = 1, 2, 3). AITCi: Treatment groups with i-times of AITC fumigation (i = 1, 2, 3). Bars with different letters indicate significant differences (*p* < 0.05). (**C**,**D**) Principal coordinate analysis (PCoA) of bacterial (**C**) and fungal (**D**) communities based on Bray–Curtis distance. Symbols correspond to the treatments described above. * 0.01 < *p* ≤ 0.05, ** 0.001 < *p* ≤ 0.01, *** *p* ≤ 0.001.

Continuous AITC fumigation exerted distinct disturbance patterns on soil bacterial and fungal communities. Analysis of alpha diversity (assessed using the Chao1 index) revealed that fungal communities exhibited greater sensitivity to AITC than bacterial communities (Figure 1A,B). The decline in their Chao1 index increased linearly with fumigation frequency, rising from 28.6% to 78.1%. In contrast, the bacterial community maintained relative stability, with a maximum decline (4.2%) much lower than that of the fungal community (78.1%), demonstrating stronger homeostasis maintenance capacity. PCoA further confirmed this trend (Figure 1C,D): AITC-treated groups were spatially separated from controls, with bacterial communities exhibiting clear succession trajectories as fumigation frequency increased. Fungal communities, however, underwent complete structural alterations after multiple fumigations, indicating that AITC exerts stronger disruptive and cumulative inhibitory effects on fungi.

### 3.2. Differentiation Characteristics of AITC-Sensitive and Tolerant Clusters

**Figure 2:**
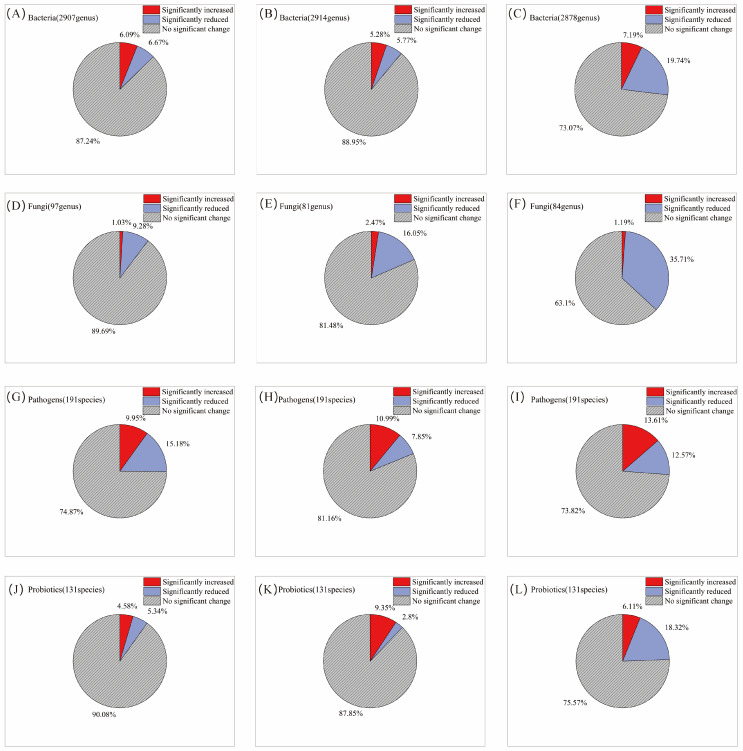
Changes in the proportion of microorganisms by category with increasing frequency of AITC fumigation. (**A**,**D**,**G**,**J**) Fumigated once; (**B**,**E**,**H**,**K**) fumigated twice; and (**C**,**F**,**I**,**L**) fumigated three times. The proportions represent the mean percentage of genera showing a significant increase or decrease relative to their respective mock-fumigated control groups (CKi).

**Figure 3:**
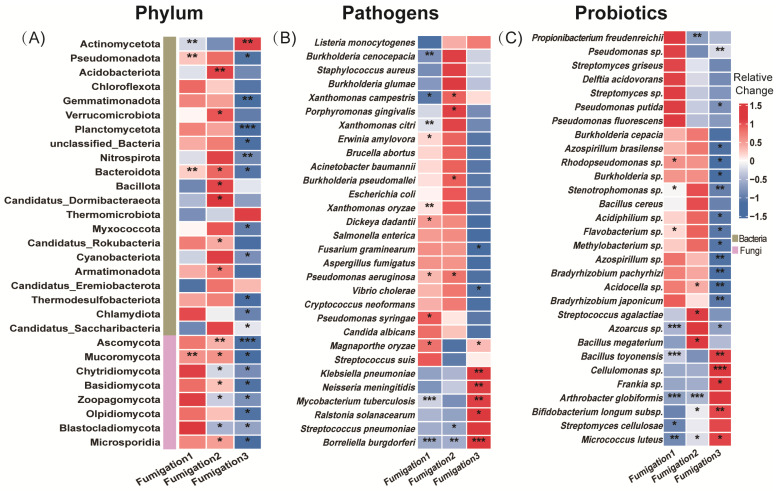
Heatmap showing microbial changes with fumigation frequency. (**A**) Phylum-level bacterial communities (top 22) and fungal communities (top 8); (**B**) top 30 plant pathogens by abundance (at the species level); and (**C**) top 30 abundant plant-beneficial microorganisms (at the species level). Relative change was calculated as (AITCi − CKi)/CKi, where CKi represents the mock-fumigated control group subjected to the same physical sealing procedure i times (i = 1, 2, 3). Asterisks denote significance levels (* *p* < 0.05, ** *p* < 0.01, and *** *p* < 0.001).

Following a single AITC fumigation, 6.1% and 6.7% of the 2907 bacterial genera were significantly increased and decreased, respectively (Figure 2A). After two fumigations, the proportions were 5.3% and 5.8%, respectively, among 2914 genera (Figure 2B). By the third fumigation, the proportion of increased genera rose slightly to 7.2%, whereas the proportion of decreased genera rose sharply to 19.7% (Figure 2C), indicating that the inhibitory effect of AITC fumigation on bacteria intensified with cumulative fumigation frequency. In contrast, fungal genera exhibited greater sensitivity to AITC. After one fumigation, the proportions of significantly increased and decreased fungal genera were 1.0% and 9.3%, respectively (Figure 2D); after two fumigations, these proportions were 2.5% and 16.1% (Figure 2E); and after three fumigations, the proportion of significantly decreased genera rose to 35.7%, while the proportion of significantly increased genera was only 1.2% (Figure 2F). This indicates that AITC exerts a significantly greater inhibitory effect than a stimulatory effect on fungi, with the inhibitory intensity increasing approximately 2.8-fold with increasing fumigation frequency. At the phylum level (Figure 3A), *Actinomycetota*, *Pseudomonadota*, *Bacteroidota*, and *Mucoromycota* exhibited high sensitivity to AITC, showing significant changes after a single fumigation, while *Chloroflexota*, *Thermomicrobiota*, and *Candidatus_Eremiobacterota* exhibited high tolerance, showing no significant changes even after three consecutive fumigations.

Among the 191 pathogenic bacterial species examined, 10% of genera were significantly increased, and 15.2% were decreased after a single AITC fumigation (Figure 2G). After two fumigations, these proportions changed to 11% and 7.9%, respectively (Figure 2H). After three fumigations, the proportion of significantly increased genera rose to 13.6%, while that of decreased genera increased to 12.6% (Figure 2I). This suggests that AITC’s inhibitory effect on pathogens gradually weakened with increasing fumigation frequency, while its stimulatory effect exhibited an upward trend. At the species level (Figure 3B), multiple plant pathogens represented by *Pseudomonas* (*P. aeruginosa*, *P. syringae*), *Xanthomonas* spp., and *Erwinia*/*Dickeya* (*E. amylovora*, *D. dadantii*) exhibited extreme sensitivity to AITC, while species such as *Salmonella enterica*, *Staphylococcus aureus*, *Escherichia coli*, *Aspergillus fumigatus*, and *Listeria monocytogenes* exhibited strong tolerance, becoming residual or dominant groups after fumigation.

Analysis of 131 beneficial bacterial strains showed that 4.6% and 5.3% of genera were significantly increased and decreased, respectively, after a single AITC fumigation (Figure 2J). After two fumigations, the proportion of increased genera rose to 9.4%, whereas that of decreased genera dropped to 2.8% (Figure 2K). Following three fumigations, the proportion of decreased genera surged to 18.3%, while that of increased genera declined to 6.1% (Figure 2L), indicating that prolonged continuous fumigation ultimately suppresses bacterial communities. Among beneficial bacteria (Figure 3C), sensitive taxa were primarily enriched in the genera *Pseudomonas*, Bacillus (e.g., *B. toyonensis*), and *Actinomyces* (*A. globiformis*). In contrast, ecologically resilient, tolerant species were concentrated in *Streptomyces* spp., specific functional *Bacillus* (*B. cereus*), *Pseudomonas fluorescens*, and *Desulfovibrio acidovorans*. This species-level selective reshaping provides a crucial basis for subsequently screening tolerant, functional bacteria for ecological remediation.

### 3.3. Isolation of AITC-Tolerant Microorganisms and Validation of Virulence Differences

**Figure 4:**
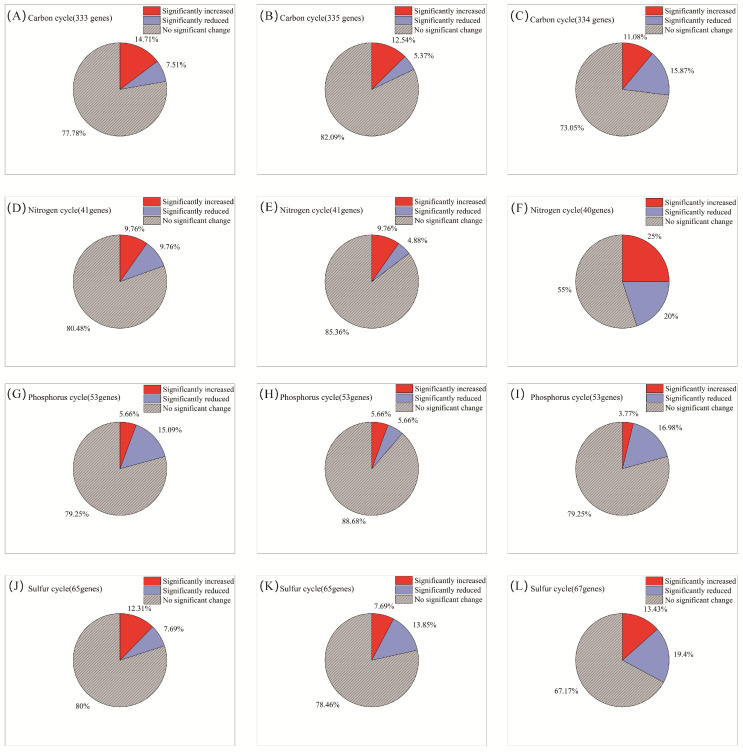
Changes in the proportion of microorganisms by category with increasing frequency of AITC fumigation. (**A**,**D**,**G**,**J**) Fumigated once; (**B**,**E**,**H**,**K**) fumigated twice; and (**C**,**F**,**I**,**L**) fumigated three times. The proportions represent the mean percentage of microbial genera showing significant changes relative to their respective mock-fumigated control groups (CKi).

**Figure 5:**
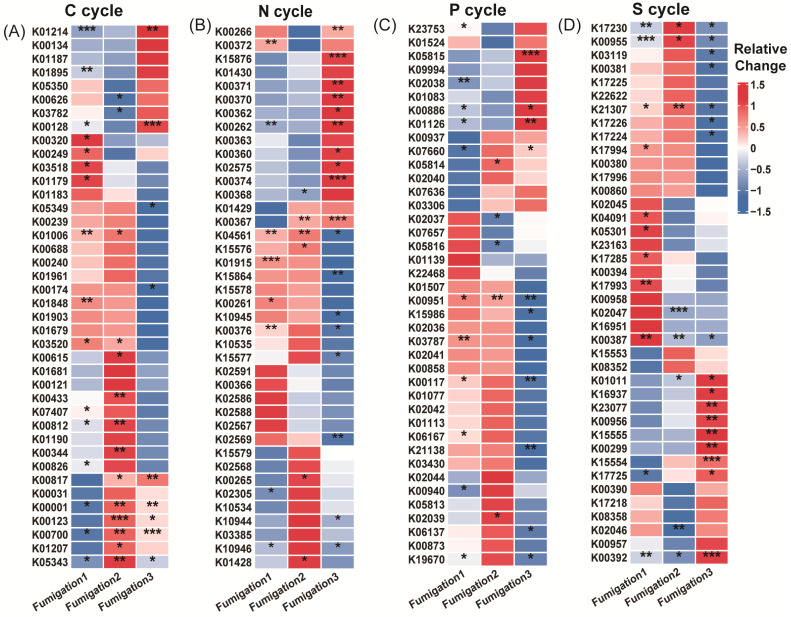
Heatmap showing functional genes across fumigation treatments. (**A**) Top 40 most abundant carbon cycle genes; (**B**) top 40 most abundant nitrogen cycle genes; (**C**) top 40 most abundant phosphorus cycle genes; and (**D**) top 40 most abundant sulfur cycle genes. Relative change was calculated as (AITCi − CKi)/CKi, where CKi represents the mock-fumigated control group subjected to the same physical sealing procedure i times (i = 1, 2, 3). Asterisks denote significance levels (* *p* < 0.05, ** *p* < 0.01, and *** *p* < 0.001).

AITC fumigation did not uniformly suppress soil functional genes. Instead, it drove directional selection from sensitive to tolerant functional communities through differential selective pressures. The selective effect of AITC on carbon cycle genes exhibited clear frequency dependence (Figure 4A–C). After one and two fumigations, the proportions of significantly altered cycle genes were 22.2% and 17.9%, respectively. At these stages, stimulatory effects (14.7%, 12.5%, respectively) substantially exceeded inhibitory effects (7.5%, 5.4%, respectively). After three fumigations, the proportion of upregulated genes decreased to 11.1%, while that of downregulated genes increased sharply to 15.9%. Correspondingly, the proportion of genes showing no significant change decreased from 77.8% to 73.1% after the third fumigation. Sensitive genes were predominantly associated with organic carbon oxidation (acd, cutL, adh, acs, and oxS), carbon fixation (ppdK and mcmA1), and polysaccharide degradation (glgB and treX) modules (Figure 5A and Table S1). In contrast, tolerant genes were highly enriched in core TCA cycle modules (acnA, icd, sdhA/B, frdB, and fumC) and fundamental metabolic support modules (malZ, glgP, gapA, and sucC). These tolerant genes maintained a steady state of “no significant change” under continuous stress, demonstrating exceptional functional robustness.

Nitrogen cycle genes exhibited high sensitivity to AITC fumigation (Figure 4D–F). The proportion of genes showing significant responses increased substantially from 19.5% after one fumigation to 27% after three fumigations. Concurrently, the proportion of genes showing no significant change decreased from 80.5% to 55%. After three fumigations, the proportion of significantly upregulated genes (25%) exceeded that of downregulated genes (20%). Sensitive nitrogen cycle genes were primarily associated with nitrification (amoC, hao), denitrification (nosZ, norB/C), and nitrogen assimilation (glnA, gdhA, nasA/C) modules. Tolerant genes were mainly enriched in nitrogen fixation (nifD/H/K), dissimilatory nitrate reduction (nrfA, napA/B), as well as in transport and cyanide degradation (nrtC/D, nasD, cynD, ureB) modules (Figure 5B; Table S1).

Phosphorus cycle genes collectively exhibited a predominantly negative sensitivity response pattern to AITC fumigation (Figure 4G–I). Both single (15.1% downregulation) and triple (17% downregulation) AITC fumigation cycles resulted in a dominant inhibitory effect on phosphorus cycle genes. After two fumigations, the proportion of steady-state genes reached 88.7%, while the stimulatory effect decreased to its lowest level (3.8%) as fumigation frequency increased. Sensitive genes were distributed across multiple functional modules, including inorganic phosphate solubilization (gcd), phosphate regulation (phoP, relA, surE), and phosphate transport/metabolism (pstA, phnP/A, ppk2). In contrast, tolerant genes were enriched in organic phosphate mineralization (phoD, phoA/B, ppx-gppA), polyphosphate metabolism (ppk1, ppk2), and core phosphorus sensing and transport (phoR, pstS/B, phnD/C) (Figure 5C; Table S1).

Sulfur cycle genes exhibited progressively reduced steady-state proportions and enhanced suppression under continuous AITC fumigation (Figure 4J–L). A single AITC fumigation primarily elicited stimulatory effects (12.3%). With increasing fumigation frequency, the proportion of steady-state genes decreased from 80% to 67.2%. After three fumigations, the proportion of downregulated genes increased to 19.4%, substantially exceeding that of upregulated genes (13.4%). Sensitive genes were predominantly associated with sulfur oxidation (sorA, soeA, and SUOX), assimilation/dissimilation sulfite reduction (sir and fccA), and organic sulfur metabolism (ssuD and ETHE1) modules. Tolerance genes were distributed across core sulfate reduction (cysD/H/J/A/C, sat, and aprA), thiosulfate reduction/sulfur oxidation complex (soxC/D, phsA, and ttrB), and organic sulfur transport (ssuA and sbp) modules (Figure 5D; Table S1).

### 3.4. AITC-Resistant Microbial Isolation and Virulence Variation Verification

Thirty bacterial strains exhibiting tolerance to AITC were isolated from triply fumigated soil using pure culture techniques. Based on 16S rRNA analysis, the isolated strains were primarily classified within the genera *Rhodococcus*, *Arthrobacter* (including *Sinomonas*), and *Bacillus* (Table 1). In vitro virulence assays further quantified tolerance differences (Table 2). The EC_50_ values of AITC for the soil-borne pathogens *R. solanacearum* and *F. oxysporum* were 1.3 mg·L^−1^ and 7.9 mg·L^−1^, respectively. In contrast, the EC_50_ for the tolerant beneficial bacterium Bacillus subtilis reached 47 mg·L^−1^. These results demonstrate that beneficial bacteria such as *Bacillus* spp. exhibit AITC tolerance thresholds 6–30 times higher than those of pathogenic bacteria, supporting their potential as pioneering strains for post-fumigation soil remediation.

## 4. Discussion

### 4.1. Differential Selection and Asymmetric Response of AITC on Soil Microbial Communities

This study demonstrates that AITC fumigation significantly restructured soil microbial communities [6,7,8], with fungi exhibiting greater vulnerability than bacteria. This disparity may originate from fundamental differences in cellular structure. Fungal cell walls, rich in chitin and glucan, are more permeable to lipophilic isothiocyanate compounds, leading to membrane integrity loss and oxidative stress [1,3]. In contrast, bacteria possess a robust peptidoglycan layer and spore-forming capabilities that provide substantial protection. Furthermore, community succession exhibied distinct niche- filtering characteristic. AITC rapidly eliminates sensitive pathogens such as *Fusarium* and *Ralstonia*, thereby releasing ecological space for metabolically diverse and stress-resistant bacteria (e.g., *Arthrobacter* and *Bacillus*) to dominate during fumigation stages. This asymmetric succession creates a temporal window for tolerant beneficial bacteria to occupy vacated niches [18].

### 4.2. Functional Screening of Key Biogeochemical Cycle Functions

Metagenomic analyses revealed that AITC disrupts soil functions selectively rather than causing uniform damage. The significant suppression of nitrogen cycling functions, particularly nitrification and denitrification, correlated with a reduced abundance of relevant functional communities within the *Pseudomonadota* phylum. This explains why fumigation often impedes the short-term soil-nitrate nitrogen accumulation [14,19]. The suppression of these key nitrogen cycling processes has direct agronomic implications. The reduced bioavailability of nitrate nitrogen—a primary nitrogen source for most crops—may necessitate adjustments in fertilizer management following AITC fumigation. To prevent temporary nitrogen deficiency during early crop growth, postponing top-dressing fertilizer application or supplementing with readily available nitrogen sources during the critical seedling stage is recommended. However, genes associated with the TCA cycle exhibited notable tolerance, suggesting robust functional redundancy within core soil metabolic networks [20,21,22], which maintains minimal metabolic activities under chemical stress. This functional screening outcome indicates that fumigated soils enter a semi-functional state, requiring targeted remediation of impaired nitrogen transformation and organic matter degradation functions.

### 4.3. Precision Microbiome Restoration Strategy Based on the “Sensitive-Tolerant” Profile

Traditional soil remediation following fumigation often relies on the indiscriminate application of broad-spectrum exogenous microbial agents, which frequently fail to establish due to poor environmental adaptability [23,24,25]. The sensitive–tolerant profile established in this study, combined with the isolated endogenous tolerant strains, provides a new paradigm for precision remediation. Given that *Bacillus subtilis* and other *Bacillus* species exhibit AITC tolerance thresholds (EC_50_ > 40 mg·L^−1^) substantially higher than those of pathogens, these strains can be applied early during the safety interval before complete fumigant degradation. Through this staggered application strategy, tolerant beneficial bacteria can preferentially colonize niches vacated by sensitive pathogens, establishing a biological barrier against pathogen resurgence [26,27]. Therefore, future efforts should focus on developing combined microbial agents that integrate AITC-tolerant strains (e.g., *Bacillus*, *Arthrobacter*) with functionally complementary microorganisms (e.g., *nitrifying* bacteria). This approach represents a shift from total eradication and replacement to precision regulation.

## 5. Conclusions

Continuous AITC fumigation substantially reshaped the soil microbiome through strong selective pressure, characterized by structural collapse of fungal communities and tolerance-driven succession of bacterial communities. Functionally, nitrogen cycling and sulfur oxidation processes were most susceptible to AITC disruption, whereas core carbon metabolism remained relatively stable. This study identified sensitive pathogenic taxa, primarily represented by *Pseudomonas* and *Fusarium*, alongside tolerant beneficial groups, notably *Bacillus* and *Arthrobacter*. Notably, *Bacillus* species exhibited high tolerance thresholds, positioning them as ideal pioneer species for reconstructing soil microbial communities after fumigation. These findings provide critical theoretical foundations and germplasm resources for establishing precise remediation timelines after AITC fumigation and developing targeted bioremediation strategies.

## Figures and Tables

**Table 1:** Types of bacteria isolated from soil after three fumigations.

Classification Status			Strains	Number of Strains
*Actinobacteria*	*Actinobacteria*	*Micrococcales*	*Sinomonas* sp.	1
			*Arthrobacter* sp.	6
			*Pseudarthrobacter phenanthrenivorans Sphe3*	11
			*Microbacterium* sp. *YJN-G*	3
			*Micrococcaceae bacterium*	2
		*Corynebacteriales*	*Rhodococcus ruber*	1
*Firmicutes*	*Bacilli*	*Bacillales*	*Bacillus* sp.	5
			*Priestia megaterium*	1

**Table 2:** Dose–response relationship of AITC on pathogenic bacteria and beneficial bacteria.

Microbial Strain	Linear Regression Equation	Coefficient of Determination R^2^	EC_50_ (mg·L^−1^)	EC_90_ (mg·L^−1^)
*R.solanacearum*	y = −0.67 + 0.26x	0.935	1.282	3.144
*F. oxysporum*	y = 3.018 − 2.704x	0.940	7.872	42.090
*F. graminearum*	y = 2.007 − 1.658x	0.965	6.699	83.307
*Trichoderma polysporum*	y = 2.604 − 2.605x	0.961	10.012	69.891
*Bacillus amyloliquefaciens*	y = −1.39 + 1.51x	0.790	8.221	238.680
*Bacillus subtilis*	y = −1.08 + 0.65x	0.817	46.945	125,609
*Sinomonas atrocyanea*	y = −6.34 + 7.04x	0.946	7.463	15.374

## Data Availability

The original contributions presented in this study are included in the article/Supplementary Materials. Further inquiries can be directed to the corresponding authors.

## Supplementary Materials

The following supporting information can be downloaded at: https://www.mdpi.com/article/10.3390/microorganisms14020345/s1, Table S1: Sensitive and tolerant gene.

## References

[1] Lin C.M., Preston J.F., Wei C.I. (2000). Antibacterial Mechanism of Allyl Isothiocyanate. J. Food Prot..

[2] Murata M., Yamashita N., Inoue S., Kawanishi S. (2000). Mechanism of Oxidative DNA Damage Induced by Carcinogenic Allyl Isothiocyanate. Free Radic. Biol. Med..

[3] Zhang C., Ma Z., Zhang X., Wu H. (2016). Transcriptomic Alterations in Sitophilus Zeamais in Response to Allyl Isothiocyanate Fumigation. Pestic. Biochem. Physiol..

[4] Dahlin P., Hallmann J. (2020). New Insights on the Role of Allyl Isothiocyanate in Controlling the Root Knot Nematode Meloidogyne Hapla. Plants.

[5] Li Y., Lu D., Xia Y., Xu X., Huang H., Mei X., Yang M., Li J., Zhu S., Liu Y. (2023). Effects of Allyl Isothiocyanate Fumigation on Medicinal Plant Root Knot Disease Control, Plant Survival, and the Soil Bacterial Community. BMC Microbiol..

[6] Fang W., Wang X., Huang B., Zhang D., Liu J., Zhu J., Yan D., Wang Q., Cao A., Han Q. (2019). Comparative Analysis of the Effects of Five Soil Fumigants on the Abundance of Denitrifying Microbes and Changes in Bacterial Community Composition. Ecotoxicol. Environ. Saf..

[7] Zhu J., Ren Z., Huang B., Cao A., Wang Q., Yan D., Ouyang C., Wu J., Li Y. (2020). Effects of Fumigation with Allyl Isothiocyanate on Soil Microbial Diversity and Community Structure of Tomato. J. Agric. Food Chem..

[8] Fang W., Yan D., Huang B., Ren Z., Wang X., Liu X., Li Y., Ouyang C., Migheli Q., Cao A. (2019). Biochemical Pathways Used by Microorganisms to Produce Nitrous Oxide Emissions from Soils Fumigated with Dimethyl Disulfide or Allyl Isothiocyanate. Soil Biol. Biochem..

[9] Cheng H., Zhang D., Huang B., Song Z., Ren L., Hao B., Liu J., Zhu J., Fang W., Yan D. (2020). Organic Fertilizer Improves Soil Fertility and Restores the Bacterial Community after 1,3-Dichloropropene Fumigation. Sci. Total Environ..

[10] Cheng H., Zhang D., Ren L., Song Z., Li Q., Wu J., Fang W., Huang B., Yan D., Li Y. (2021). Bio-activation of Soil with Beneficial Microbes after Soil Fumigation Reduces Soil-Borne Pathogens and Increases Tomato Yield. Environ. Pollut..

[11] Li Q., Zhang D., Cheng H., Ren L., Jin X., Fang W., Yan D., Li Y., Wang Q., Cao A. (2022). Organic Fertilizers Activate Soil Enzyme Activities and Promote the Recovery of Soil Beneficial Microorganisms after Dazomet Fumigation. J. Environ. Manag..

[12] Fang W., Huang B., Sun Y., Yan D., Li Y., Bruno T., Roncada P., Wang Q., Cao A. (2024). Soil Amendments Promoting Nitrifying Bacteria Recovery Faster Than the Denitrifying Bacteria at Post Soil Fumigation. Sci. Total Environ..

[13] Li Q., Andom O., Fang W., Yan D., Li Y., Wang Q., Jin X., Cao A. (2024). Effects of Soil Amendments on Soil Properties, Soil-Borne Pathogens, and Strawberry Growth after Dazomet Fumigation. Agriculture.

[14] Fang W., Tian W., Yan D., Li Y., Cao A., Wang Q. (2025). Linkages Between Soil Nutrient Turnover and Above-ground Crop Nutrient Metabolism: The Role of Soil Microbes. iMetaOmics.

[15] Tian W., Wang Q., Cao A., Yan D., Li Y., Liu Z., Yang B., Fang W. (2025). Genotype-dependent Responses of Pepper Endophytes to Soil Microbial Community Shifts Induced by Allyl Isothiocyanate Fumigation. Front. Microbiol..

[16] Zhang S., Liu X., Jiang Q., Shen G., Ding W. (2017). Legacy Effects of Continuous Chloropicrin-Fumigation for 3-Years on Soil Microbial Community Composition and Metabolic Activity. AMB Express.

[17] Sun G., Zhang Q., Dong Z., Dong D., Fang H., Wang C., Dong Y., Wu J., Tan X., Zhu P. (2022). Antibiotic Resistant Bacteria: A Bibliometric Review of Literature. Front. Public Health.

[18] Ruzo L.O. (2006). Physical, Chemical and Environmental Properties of Selected Chemical Alternatives for the Pre-Plant Use of Methyl Bromide As Soil Fumigant. Pest Manag. Sci..

[19] Yan D., Wang Q., Mao L., Li W., Xie H., Guo M., Cao A. (2013). Quantification of the Effects of Various Soil Fumigation Treatments on Nitrogen Mineralization and Nitrification in Laboratory Incubation and Field Studies. Chemosphere.

[20] Harris J. (2009). Soil Microbial Communities and Restoration Ecology: Facilitators or Followers?. Science.

[21] Zhao Z., He J., Geisen S., Han L., Wang J., Shen J., Wei W., Fang Y., Li P., Zhang L. (2019). Protist Communities Are More Sensitive to Nitrogen Fertilization Than Other Microorganisms in Diverse Agricultural Soils. Microbiome.

[22] Dai T., Wen D., Bates C.T., Wu L., Guo X., Liu S., Su Y., Lei J., Zhou J., Yang Y. (2022). Nutrient Supply Controls the Linkage Between Species Abundance and Ecological Interactions in Marine Bacterial Communities. Nat. Commun..

[23] Shen Z., Xue C., Penton C.R., Thomashow L.S., Zhang N., Wang B., Ruan Y., Li R., Shen Q. (2018). Suppression of Banana Panama Disease Induced by Soil Microbiome Reconstruction Through an Integrated Agricultural Strategy. Soil Biol. Biochem..

[24] Liu F., Mao J., Kong W., Hua Q., Feng Y., Bashir R., Lu T. (2020). Interaction Variability Shapes Succession of Synthetic Microbial Ecosystems. Nat. Commun..

[25] Gu Y., Banerjee S., Dini-Andreote F., Xu Y., Shen Q., Jousset A., Wei Z. (2022). Small Changes in Rhizosphere Microbiome Composition Predict Disease Outcomes Earlier Than Pathogen Density Variations. ISME J..

[26] Wei Z., Gu Y., Friman V., Kowalchuk G.A., Xu Y., Shen Q., Jousset A. (2019). Initial Soil Microbiome Composition and Functioning Predetermine Future Plant Health. Sci. Adv..

[27] Wen T., Xie P., Penton C.R., Hale L., Thomashow L.S., Yang S., Ding Z., Su Y., Yuan J., Shen Q. (2022). Specific Metabolites Drive the Deterministic Assembly of Diseased Rhizosphere Microbiome Through Weakening Microbial Degradation of Autotoxin. Microbiome.

